# Transcriptome sequencing reveals signatures of positive selection in the Spot-Tailed Earless Lizard

**DOI:** 10.1371/journal.pone.0234504

**Published:** 2020-06-15

**Authors:** Jose A. Maldonado, Thomas J. Firneno, Corey E. Roelke, Nathan D. Rains, Juliet Mwgiri, Matthew K. Fujita

**Affiliations:** 1 Department of Biology, Amphibian and Reptile Diversity Research Center, The University of Texas at Arlington, Arlington, TX, United States of America; 2 Texas Parks and Wildlife Department, Austin, Texas, TX, United States of America; Senckenberg am Meer Deutsches Zentrum fur Marine Biodiversitatsforschung, GERMANY

## Abstract

The continual loss of threatened biodiversity is occurring at an accelerated pace. High-throughput sequencing technologies are now providing opportunities to address this issue by aiding in the generation of molecular data for many understudied species of high conservation interest. Our overall goal of this study was to begin building the genomic resources to continue investigations and conservation of the Spot-Tailed Earless lizard. Here we leverage the power of high-throughput sequencing to generate the liver transcriptome for the Northern Spot-Tailed Earless Lizard (*Holbrookia lacerata*) and Southern Spot-Tailed Earless Lizard (*Holbrookia subcaudalis*), which have declined in abundance in the past decades, and their sister species, the Common Lesser Earless Lizard (*Holbrookia maculata*). Our efforts produced high quality and robust transcriptome assemblies validated by **1**) quantifying the number of processed reads represented in the transcriptome assembly and **2**) quantifying the number of highly conserved single-copy orthologs that are present in our transcript set using the BUSCO pipeline. We found 1,361 1-to-1 orthologs among the three *Holbrookia* species, *Anolis carolinensis*, and *Sceloporus undulatus*. We carried out dN/dS selection tests using a branch-sites model and identified a dozen genes that experienced positive selection in the *Holbrookia* lineage with functions in development, immunity, and metabolism. Our single-copy orthologous sequences additionally revealed significant pairwise sequence divergence (~.73%) between the Northern *H*. *lacerata* and Southern *H*. *subcaudalis* that further supports the recent elevation of the Southern Spot-Tailed Earless Lizard to full species.

## Introduction

The continuing reduction of per-base costs of high-throughput sequencing (HTS) methodologies has provided new opportunities to generate large-scale genomic and transcriptomic resources. This is especially beneficial for data deficient, non-model organisms that may require conservation action, are commercially valuable, or are excellent systems to investigate ecological and evolutionary questions [1–4]. The field of conservation genetics has historically relied on the use of few dozen genetic markers (allozymes, microsatellites, mitochondrial DNA, and a few nuclear genes) to understand population structure, viability, and evolutionary processes (e.g. genetic drift, selection, and migration) [5,6]. With the low cost of HTS, it is now possible to rapidly generate hundreds to thousands of genetic markers from multiple individuals and populations to assay genetic diversity for virtually any species [7]. These larger datasets can overcome some of the limitations of traditional methods used in conservation genetics that yield only a few variable loci [8]. For example, with hundreds to thousands of variable sites, HTS datasets can be beneficial for species management by providing high resolution and accurate inferences of important parameters such as genetic diversity, inbreeding depression, effective population size [9,10], as well as historical demography and local adaptations. All of this can provide insight into resolving taxonomic uncertainties to determine which species need immediate attention and protection [8,11]. Using RNA-Seq methodologies to address the types of questions for species with no available genomic resources is becoming increasingly favorable [12–14]. As long as appropriately preserved tissue is available, it is possible to sequence the expressed genes by sequencing complementary DNA (cDNA) libraries. By using millions of short reads generated by massive parallel sequencing of cDNA libraries and robust assembly methods [15,16], one can generate a high coverage *de novo* transcriptome assembly without the need for a reference genome. Because transcriptome sequencing is versatile, it is a desirable option for developing conservation genetics tools because it largely circumvents the time-consuming process of identifying and optimizing genetic markers (e.g. primer development and testing) [12,17]. Furthermore, transcriptome sequencing captures protein coding regions with functional significance [2,18]. The usage of gene expression data for conservation biology is an emerging field that will significantly benefit wildlife management [19].

Our focus in this paper is the development of genomic resources for the Northern and Southern Spot-tailed Earless Lizard (*Holbrookia lacerata* and *Holbrookia subcaudalis)*, whose historic range extends from central to southern Texas, and into northeastern Mexico. For the past several decades, *H*. *lacerata* has faced taxonomic uncertainties and confusion. Initially, there was a single species, *H*. *lacerata*, that included the currently-recognized *H*. *lacerata* as well as its sister species, *H*. *maculata* [20]. *Holbrookia lacerata* was then reduced to a subspecies under *H*. *maculata* [21], and subsequently elevated back to full species status [22]. Subsequently, two subspecies of *H*. *lacerata* were recognized, *H*. *l*. *lacerata* and *H*. *l*. *subcaudalis*, that are geographically separated by the Balcones Escarpment fault line. Renewed interest in resolving their taxonomic status revealed that the disjunct populations are reciprocally monophyletic with an approximately 4% mitochondrial sequence divergence [3]. Most recently, Hibbits *et al*. (2019) took an integrative approach and used morphology, mitochondrial, and nuclear data to elevate each subspecies to full species status [23].

Both species have experienced a sharp decline in distribution and abundance through their historical range and are labeled as near threatened by the International Union for Conservation of Nature organization. The most notable decrease has occurred in southern Texas, where *H*. *subcaudalis* populations have become increasingly fragmented and isolated, with fewer observations being made throughout its historic range. Hypotheses for the decline of *H*. *lacerata* include pesticides and agriculture practices [24], and urbanization, exotic grasses, and invasive fauna are additional factors contributing to their decline [25]. The conservation concerns of both species have led to recent studies utilizing *H*. *lacerata* as a focal organism for better land management practices in Texas [25–27], and efforts are being made to protect both species under the Endangered Species Act [28].

The decline in *H*. *lacerata* and *H*. *subcaudalis* abundance can have a substantial impact on population viability. Small and fragmented populations can lead to an increase in homozygosity, the disappearance of genetic diversity, and an increase in the frequency deleterious variants become fixed can lead to inbreeding depression, and thus a reduction in individual fitness. The goal of this study is to provide a detailed characterization of a transcriptome as a means to generate molecular resources for conservation studies of *H*. *lacerata* and *H*. *subcaudalis*. We provide an annotated liver transcriptome, identify adaptive loci, and estimate genetic distance, all of which are of value for the conservation efforts of the North and Southern Spot-tailed Earless Lizard.

## Materials and methods

### Sampling

Samples for this study were collected in summer 2015 from three different localities. Lizards were caught by hand or loops and humanely euthanized under our IACUC protocol (#A16.010) approved by the University of Texas at Arlington. We harvested skeletal muscle, liver, heart, blood, and integument from one individual per species and stored the tissue in RNAlater (Sigma-Aldrich, St. Louis, MO). We preserved the specimens with 10% formalin and deposited them in the Amphibian and Reptile Diversity Research Center (ARDRC) at the University of Texas at Arlington (see S1 Table for locality data and field numbers).

### RNA extraction and RNA-Seq library preparation for sequencing

Total RNA was extracted from liver tissue using a Promega SV Total RNA Isolation kit (Promega, Madison, WI) following the manufacturer’s protocol. We quantified our RNA extractions on the Qubit 2.0 Fluorometer (Life Technologies, Carlsbad, CA), and assessed RNA quality and size distribution on an Agilent 2100 Bioanalyzer (Agilent Technologies, Santa Clara, CA). All RNA extractions yielded high-quality RNA, with all samples having an RNA integrity number (RIN) >8. RNA isolates from each sample were sent to the Brigham Young University DNA Sequencing Center to generate cDNA libraries using the Kapa Biosystems RNA depletion kit (Kapa Biosystems, Wilmington, MA) and sequenced on the Illumina HiSeq 2500 (Illumina, San Diego, CA) generating 100bp paired-end sequences.

### *De novo* transcriptome assembly and quality assessments

The data were processed to remove low quality reads using Trimmomatic v.32 [29]. We used the following parameters to trim and remove failed reads: a 4-base sliding window trimming nucleotides with a Q score <5 and discarding reads <25bp long [30]. To ensure successful quality trimming and filtering, we ran the processed reads through FASTQC v0.10.1 (Babraham Bioinformatics) to evaluate read quality, length, and the number of reads retained. With no reference genomes for any *Holbrookia* species and given that previous results have demonstrated that guided transcriptome assembly methods for diverged species typically perform worse than *de novo* assembly [31,32], we carried out *de novo* assemblies using Trinity short-read assembler V2.2.1 [15] for each sample.

**Fig 1:**
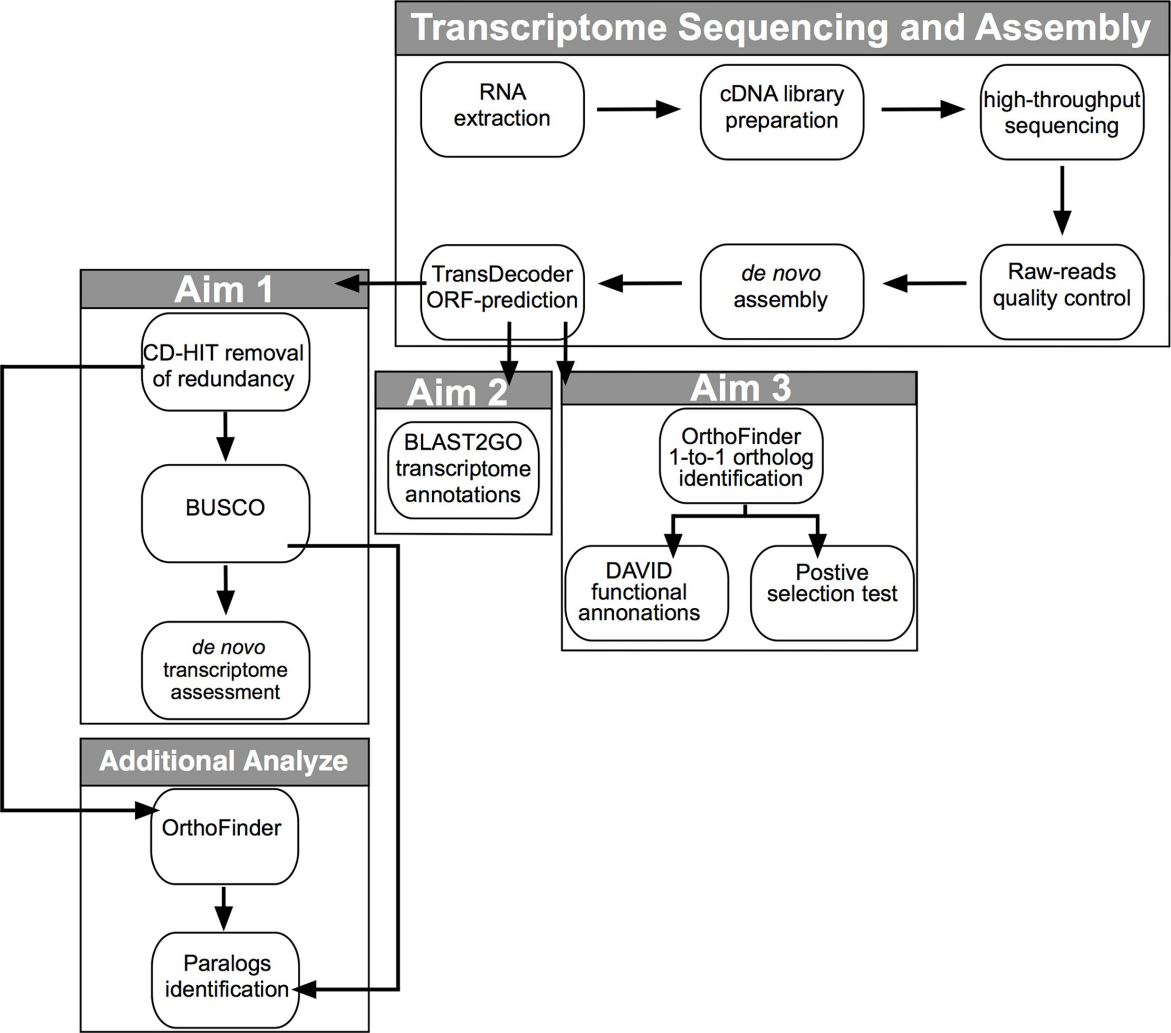
Schematic overview of our workflow to assemble the transcriptome of *H*. *lacerata*, *H*. *subcaudalis*, and *H*. *maculata*, and analysis carried out in this study.

To measure the completeness of our assembled transcripts, we performed two quality assessments as suggested by the Trinity package. First, to evaluate the assembly quality of each transcriptome, we mapped the input processed RNA-Seq reads back to their corresponding assemblies for each species of *Holbrookia* and quantified the number of input reads represented in our *de novo* transcriptome assemblies. Reads were mapped back to the transcriptome using the short read aligner Bowtie 2 v2.3.4 using the–local and–no-unal options [33]. Second, we ran our *Holbrookia* protein coding transcript set (see below for protein coding transcript set generation) through CD-Hit v4.8.1 (90% sequence identity threshold with all other parameters set to default) [34] to produce a non-redundant protein coding transcript set for each individual. We compared our non-redundant transcripts with a set of 3,950 highly conserved single-copy tetrapod orthologs using the BUSCO (benchmarking universal single-copy orthologs) v3 pipeline [35]. Fig 1 is an overview of our transcriptome assembly and analysis pipeline described below.

### Identification of orthologs and paralogs and pairwise distance

To identify candidate coding genes from our assembled transcript sets with the longest open reading frames (ORFs), we used the TransDecoder v3.0.1 pipeline [36]. To maximize the number of ORFs captured and to ensure we did not lose any potential coding genes, we ran TransDecoder optional homology search against the PFAM database [37]. We ran the TransDecoder pipeline on our three *de novo Holbrookia* transcriptomes, the *Anolis carolinensis* transcriptome downloaded from NCBI server (GCF_000090745.1), and the publicly available *Sceloporus undulatus* transcriptome [38].

We used OrthoFinder v2.2.3to identify orthologous genes between all five species from the protein coding transcript set generated by TransDecoder. OrthoFinder uses a BLAST all-vs-all algorithm and performs reciprocal best-hit BLAST searches that normalize the bit scores to overcome transcript length bias in the ortholog detection [39]. We extracted all of the 1-to-1 orthologs found between all five species into Fasta files and performed an amino-acid guided alignment for each ortholog using MACSE v1.2 [40]. We inspected our multiple species codon alignments and removed ten orthologs for having a poor alignment. We used Geneious R9 (https://www.geneious.com) to calculate the uncorrected pairwise distance across the remaining orthologous alignments.

To identify paralogs (duplicated single-copy orthologs) in our *Holbrookia* transcripts, we used our non-redundant protein coding transcript set generated by CD-Hit to reduce false positive detection of paralogs. We searched against the tetrapod single-copy ortholog database to determine the number of transcripts that are likely paralogs using the BUSCO pipeline [35] and OrthoFinder all-vs-all BLAST [39] algorithm.

### DAVID functional analysis

We submitted all *A*. *carolinensis* gene IDs from the 1-to-1 orthologs set to the bioinformatics database DAVID [41] to identify genes in our orthologous set, cluster these genes into their biological processes, and their inclusion in biological pathways. We ran DAVID using all default parameters for enrichment and pathway analysis.

### Selection test

To identify positive selection in *Holbrookia*, we estimated dN/dS in all 1-to-1 orthologous genes found by OrthoFinder across all five individuals by following the recommendations of Yang *et al*. (2006) [42]. We used the branch-sites model (CODEML: M2 and NSites2) from the PAML v4.9 package [43], which requires an *a priori* phylogenetic tree to test for positive selection in foreground branches. We utilized a phylogenetic tree generated by OrthoFinder, by reconciling over a thousand gene trees into a species tree and designated the *Holbrookia* lineage as the foreground branches. The remaining *Anolis* and *Sceloporus* branches on the species tree were labeled as background branches. We compared the likelihood of two different models for each orthologous gene: **1)** our alternative model that allows for a proportion of the sites to be under positive selection (ω_2_ ≥ 1) along the *Holbrookia* branch, and the background branches having a proportion of sites being under purifying selection (ω_1_<1) or neutrally evolving (ω_0_ = 1); and **2)** the simplistic null model that has the ω_2_ fixed at 1 on the foreground branches, and all other branches having ω_0_ = 1 and ω_1_<1 [43,44]. We obtained likelihood values after running each transcript under two different models and carried out likelihood ratio tests (2×ΔlnL) between the models to evaluate whether the alternative model outperforms the null model. We performed a Bonferroni correction to account for multiple comparisons. A significant result from the branch-sites model is indicative that a subset of the sites in the coding gene has gone through episodic positive selection, with the selected sites providing an advantage to *Holbrookia* lineage.

### Gene annotation

We used the Blast2Go v5 pipeline [45] to annotate and assign functions to our complete protein coding gene set produced by TransDecoder: 34,214 transcripts for *H*. *maculata*, 33,379 transcripts for *H*. *lacerata*, and 29,149 transcripts for *H*. *subcaudalis*. We used the Blastp function to blast our transcript set against the NCBI NR-protein database using an e-value cutoff of 1e-5 and all other parameters set to default. Annotation was performed using an e-value cutoff of 1e-3, an annotation score of 45, and a GO weight of 5. To generate biological processes, molecular functions, and cellular components graphs we first grouped our Go annotation into GO-slim terms to simplify the input and filtered out nodes containing >10 sequences cellular component, nodes containing >10 sequences were filtered out.

## Results

### Sequencing and *De novo* transcriptome assembly

Our sequenced cDNA libraries on the Illumina Hi-Seq 2500 yielded a total of 223 million paired-end reads between all three samples, and we retained 99.5% of our reads after filtering out low-quality reads from our raw data set. FastQC verified that only high-quality reads with a Q score >30 were kept and assembled by the short-read assembler Trinity. The total number of assembled transcripts for *H*. *maculata*, *H*. lacerata, and *H*. *subcaudalis* are respectively 107,863, 99,821, and 91,278. The average maximum transcript length between all individuals was ~18,340 bp. Assembly statics for all three *Holbrookia* samples used in this study are listed in Table 1.

**Table 1:** Assembly statistics for *H*. *maculata*, *H*. *lacerata*, and *H*. *subcaudalis*.

Transcriptome sequencing and assembly statistics	*H*. *maculata*	*H*. *lacerata*	*H*. *subcaudalis*
**Raw paired-end reads**	93,688,798	73,668,784	66,629,872
**Cleaned paired-end reads**	93,464,743	73,492,888	66,508,242
**N50**	2,407	2,288	2,056
**Number of Transcripts**	107,385	99,821	91,278
**Max Transcript length**	17,863 bp	20,377 bp	16,777 bp
**Min Transcript length**	224 bp	224 bp	224 bp
**Mean Transcript length**	1,061 bp	1,037 bp	953 bp
**GC content**	43%	43%	42%

### Assembly quality assessment

Our first assembly quality check involved mapping the processed reads with Bowtie2 back to their assembled transcriptome. Across all samples, an average of 97% of the reads mapped back to their respective assemblies. If ≥70% of the input reads mapped back to their assembly, it is indicative of a robust transcriptome assembly by Trinity [15]. To further quantify the completeness of our *Holbrookia* transcript set, we ran our protein coding gene set for each individual through CD-HIT to remove redundant transcripts from the assemblies. Our *Holbrookia* protein coding transcript set (96,742) decreased by a factor of one-third to generate a non-redundant transcript list of 63,957 sequences. We ran our three non-redundant *Holbrookia* transcript sets against a conserved set of 3,950 universal tetrapod single-copy orthologs using the BUSCO pipeline [35,46]. We recovered 60–70% complete and 8–11% partial orthologs from the BUSCO tetrapod database. Between 831 to 1,113 orthologs were classified as missing from our transcript set. The high number of complete conserved single-copy orthologs present in each transcript set is indicative of high coverage and high-quality protein coding transcriptome assemblies for each Holbrookia species. All BUSCO statistics are detailed in Table 2.

**Table 2:** Benchmarking Universal Single-Copy Orthologs (BUSCO) summary of complete, duplicated, fragmented, and missing orthologs search against the 3950 single-copy orthologs.

BUSCO statistics	*H*. *maculata*	*H*. *lacerata*	*H*. *subcaudalis*
**Complete Buscos**	2,764	2,727	2,372
**Complete-single-copy BUSCOs**	2,627	2,604	2,259
**Complete-duplicated BUSCOs**	137	123	113
**Fragmented BUSCOs**	345	392	465
**Missing BUSCO**	841	831	1,113

### Orthologs and paralogs identification

**Fig 2:**
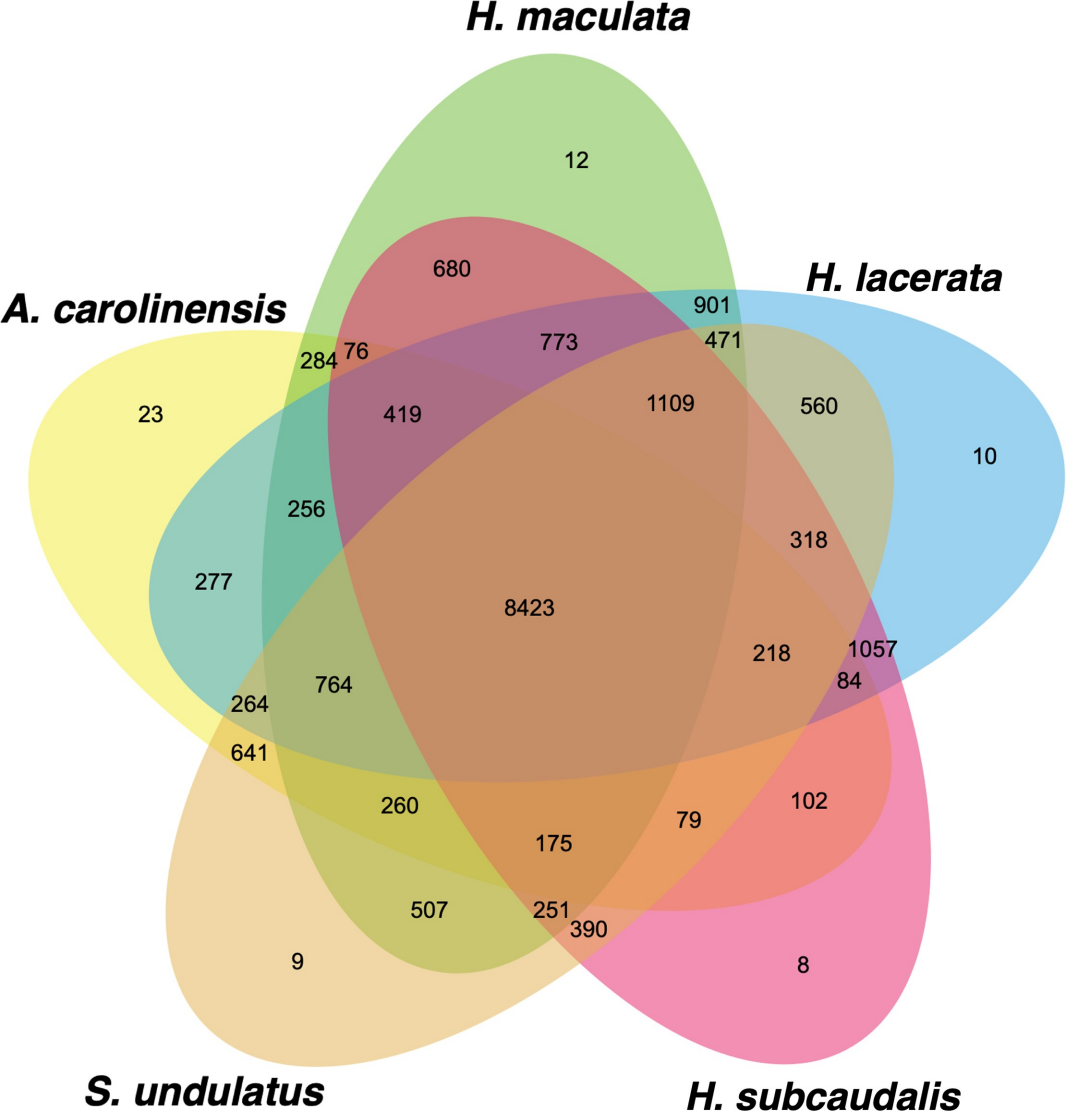
Venn diagram showing the number of shared orthologous groups identified by OrthoFinder between all four species assembled transcriptome.

To identify the 1-to-1 orthologous groups among our coding genes identified by TransDecoder between the three *Holbrookia* species, *Anolis carolinensis*, and *Sceloporus undulatus*, we ran all five individuals through OrthoFinder. TransDecoder found between 29,149 to 35,594 protein coding genes with the longest open reading frame from our assembled transcript set. We submitted a total of 160,639 coding transcripts to OrthoFinder to identify orthologous groups. OrthoFinder identified 19,401 orthogroups (defined as containing both orthologs and paralogs) containing 125,845 transcripts. We found that 43% (8,243) of the orthogroups had all five individuals present, and 56% (10,916) of the total orthogroups had at least two individuals present (Fig 2). We found 6,805 transcripts (1,361 orthologs) that were true 1-to-1 orthologs between all five individuals, and a low proportion of our transcripts that were unique to their given transcriptome assemblies.

To identify paralogs in our *Holbrookia* transcriptome, we ran our protein coding set through CD-Hit [34] to remove redundancy in our transcript set for each of our *Holbrookia* samples. BUSCO found that between 3% to 3.5% of the 3950 single-copy tetrapod orthologs that we searched against were detected as duplicated in our transcript assemblies. Our second method to identify paralogs in the *Holbrookia* transcriptome was using the OrthoFinder BLAST algorithm only on our *Holbrookia* protein coding gene list, by counting the number of self-BLAST hits identified between each transcript. We discovered 4590 transcripts in *H*. *maculata*, 4797 transcripts in *H*. *lacerata*, and 4271 transcripts in *H*. *subcaudalis* as paralogous transcripts within each species.

### DAVID functional analysis and selection test

DAVID clustered our orthologous genes into separate biological processes, 409 genes into metabolic processes, 107 genes associated with stress response processes, 190 genes that play a role in gene expression, and 216 genes that are involved in cellular component organization. DAVID could not associate 838 genes with any biological process as they did not meet the enrichment threshold (P-values < .1). DAVID identified 156 genes involved in different metabolic pathways (purine/pyrimidine metabolism, amino acid metabolism, and the Citric acid cycle). DAVID identified thirteen genes associated with the nucleotide excision repair pathway, six genes linked to nucleotide mismatch repair mechanism, and seven genes that have a role in DNA replication.

To determine if any of the 1-to-1 orthologous genes have undergone positive selection in *Holbrookia*, we carried out selection tests using PAML [44] and performed likelihood-ratio tests to assess significance. We adjusted our P-values by performing a Bonferroni correction test to account for multiple comparisons and committing a type I error. We found twelve genes from our ortholog set that have undergone positive selection with a significance value of < .05, after adjusting our P-values. Table 3 has a complete list of all twelve genes with the alternative and null likelihood values and the Bayes Empirical Bayes (BEB) score for sites under selection.

**Table 3:** The alternative and null model likelihood values for the twelve genes that show footprints of positive selection in the *Holbrookia* lineage.

Gene	Alternative Log Likelihood	Null Log Likelihood	Unadjusted P-Values	Adjusted P-Values	BEB scores for positive sites
***BNIP3L***	-1236.0192	-1245.697904	1.08E-05	7.70E-04	N 0.996
					N 0.986
					N 0.986
***TBL2***	-3224.401186	-3232.500738	5.70E-05	4.39E-03	P 0.939
					Q 0.938
***RP2***	-2022.830549	-2036.859674	1.18E-07	9.09E-06	G 0.975
					E 0.950
					E 0.995
***PHGDH***	-3332.16336	-3342.054215	8.68E-06	6.68E-04	Q 0.980
					A 0.921
***KRT18***	-2900.33107	-2907.152926	0.00022098	1.69E-02	G 0.947
					S 0.942
					N 0.977
***LURAP1***	-1161.470425	-1170.150278	3.09E-05	2.38E-03	P 0.861
					A 0.867
***CDO1***	-1337.215619	-1344.793392	9.90E-05	7.62E-03	Q 0.925
***LBHD2***	-761.2579	-788.429241	1.68E-13	1.29E-11	Q 0.988
					I 0.999
					C 0.999
					V 0.999
					D 0.999
					A 0.993
					C 0.994
					T 0.999
***GRSF1***	-2724.823688	-2731.69004	0.000210753	1.62E-02	T 0.914
					Q 0.970
***SLC9A3R1***	-1661.883638	-1669.365305	0.000109621	8.47E-03	E 0.975
***RNF81***	-1070.037763	-1079.230221	1.80E-05	1.54E-03	P 0.972
					S 0.985
***MPHOSPH6***	-1077.189311	-1086.384783	1.80E-05	1.39E-03	A 0.921
					R 0.953
					P 0.951

### Pairwise distance

We calculated the uncorrected pairwise distance using the complete single copy ortholog set. The most significant sequence divergence was found between *A*. *carolinensis* and all other species being around ~11%. The split between *Anolis* (family Dactyloidae) and the four other species in the family Phrynosomatidae occurred ~72MYA and accounts for the vast amount of genetic divergence [47]. There is an average 5.57% sequence divergence between *S*. *undulatus* and all *Holbrookia* species, with the pairwise distance between *H*. *maculata* and *H*. *lacerata* and *H*. *subcaudalis* is ~1.5%. The genetic distance between *H*. *lacerata* and *H*. *subcaudalis* 0.73% (Table 4).

**Table 4:** The uncorrected pairwise distance calculated across all orthologous gene sets between all species.

Pairwise Distance	*A*. *carolinensis*	*S*. *undulatus*	*H*. *maculata*	*H*. *lacerata*	*H*. *subcaudalis*
***A*. *carolinensis***	0				
***S*. *undulatus***	11.29%	0			
***H*. *maculata***	11.10%	5.58%	0		
***H*. *lacerata***	11.27%	5.56%	1.53%	0	
***H*. *subcaudalis***	11.29%	5.57%	1.50%	0.73%	0

### Annotations

**Fig 3:**
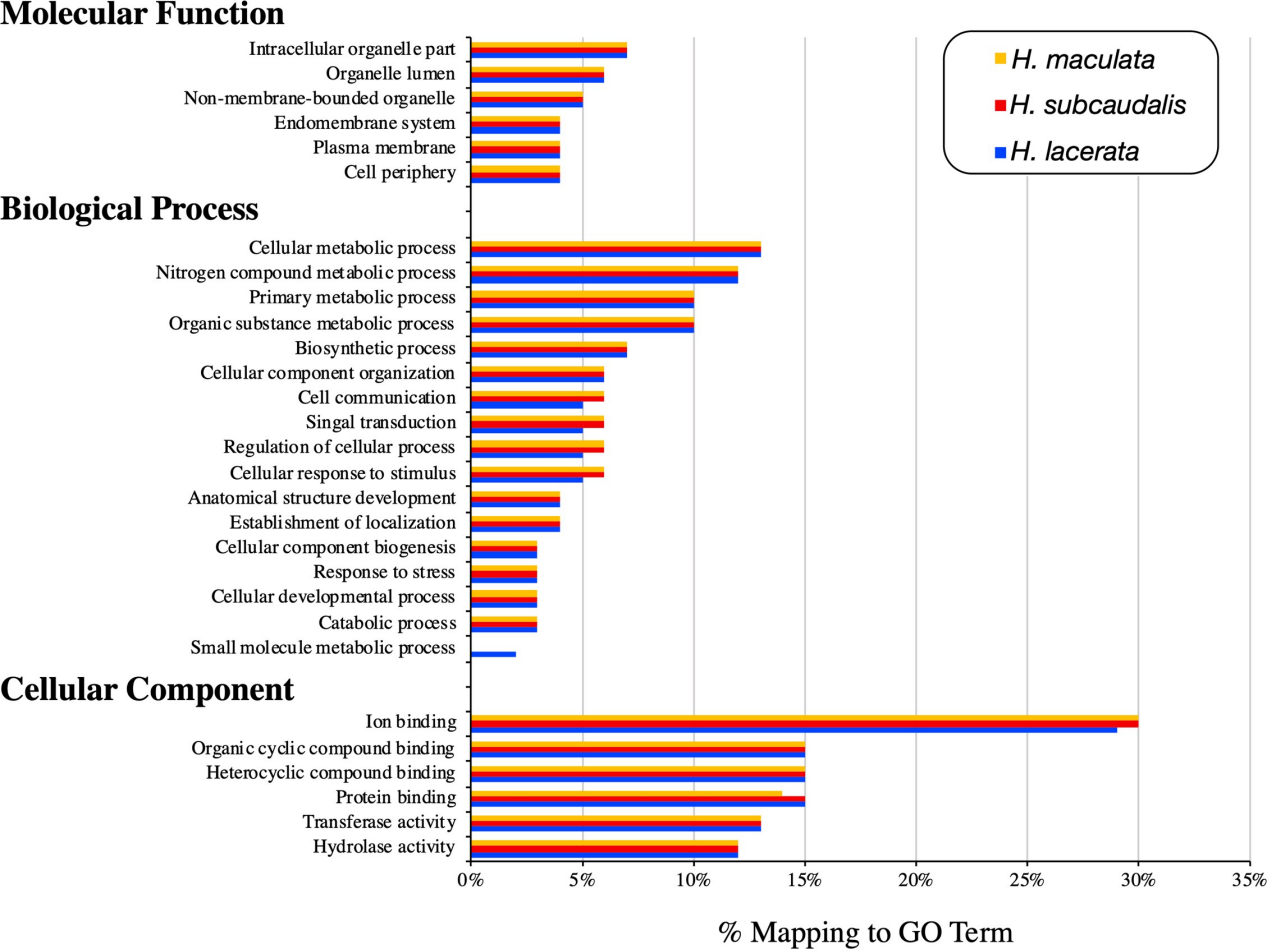
Gene ontology annotations for the liver transcriptome of *H*. *maculata* (yellow), *H*. *subcaudalis* (red), *and H*. *lacerata* (blue).

Out of the 96,742 transcripts submitted to BLAST2GO, around ~85% were successfully blasted against the NCBI NR-protein database, with the top BLAST hit being *A*. *carolinensis* with the remaining orthologs blasting to other reptile species (e.g. *Python bivittatus*, *Gecko japonicus*). We successfully annotated a total of 71,314 transcripts into 55 functional categories using GO-Slim assignments within the three categories of the GO classification system biological process, cellular component, and molecular function. The primary categories our coding genes were clustered into are cellular metabolic processes (30,145), nitrogen compound metabolic process (28,816), primary metabolic process (23,249), and ion binding (21,228). A breakdown of GO terms for each category for each *Holbrookia* sample is shown in Fig 3.

## Discussion

Genetic data are extremely useful in conservation studies because they can provide estimates of genetic diversity, population size (and viability), and local adaptations for imperiled populations and species. However, many taxa of conservation significance have little to no genetic data available, impeding studies that can inform conservation planning and action. The speed and ease of generating molecular data for the use of species management and policy have quickly increased with the transition to HTS technologies [8,48]. The decline of Northern and Southern Spot-Tailed Earless Lizard led us to generate and profile the liver transcriptome for each species. By doing so, we establish a genomic resource for future genome-scale studies on *Holbrookia* as well as provide initial insights into the divergence and evolution of *H*. *lacerata* and *H*. *subcaudalis*. Because divergence and adaptation are important components of conservation genetics, we (1) quantify the genetic distance between *H*. *lacerata* and *H*. *subcaudalis*, and (2) identify potential genes under selection in *H*. *lacerata* and *H*. *subcaudalis*.

### Transcriptome annotation

Our gene ontology analyses revealed that the biological functions of our liver transcripts closely follow similar patterns of other liver transcriptomes in squamates [49]. A large number of genes expressed in the liver play a role in metabolism, proteolysis, and nitrogen compound synthesis and breakdown. Oxidative damage can lead to DNA lesions and strand breaks, and are caused by endogenous reactive oxygen species produced during normal cellular metabolism [50]. Here we found both heath shock and glutathione peroxidase proteins in our transcriptome set that are well documented as having roles in protecting against oxidative damage [51].

### Pairwise distances

The U.S. Fish and Wildlife Service (FWS) is currently reviewing if *H*. *lacerata and H*. *subcaudalis* require protection under the endangered species act [28]. Using their historical classification as subspecies, the FWS can place either the Northern or Southern Spot-Tailed Earless Lizard in need of protection, or both. Here we have found significantly more divergence (0.73%) between the Northern and Southern Spot-Tailed Earless Lizard using conserved orthologous sequences than previous study has estimated between two allopatric species using nuclear genes [52]. The results in this study, alongside Roelke *et al*. (2018) finding that both the northern and southern lizards are genetically distinct using mitochondrial genomes, all further support the Hibbitts *et al*. (2019) recent reclassification of their taxonomy. The elevation of the Northern and Southern Spot-Tailed Earless Lizard from subspecies to full species status requires the FWS consider them separately for protection, as each species faces unique threats [23,24]. The southern *H*. *subcaudalis* has two isolated populations that were once part of a larger distribution across southern Texas, while the more robust northern species has more observations in multiple surveys of the region [23].

### DAVID analysis of orthologs and positive selection

Established methods to find signatures of adaptive evolution across the genome coupled with the increased number of molecular markers captured with HTS techniques are facilitating the discovery of adaptive variation. Identifying populations from at-risk species that show signs of local adaptive genetic variation and subsequently maintaining it are essential parts of any conservation strategy [53]. Adaptive variation can inform conservation managers on how to perform genetic rescues or assisted gene flow to raise the fitness of small populations that lack genetic diversity [54]. As the use of large scale genomic data becomes more widely adopted in the field of conservation biology, it will have a significant role in conservation management and policy.

The bulk of nonsynonymous mutations are generally considered deleterious as they alter protein structure and function, which can reduce an organism's fitness, and purifying selection should remove these mutations. Genes such as *RAD23* protein [55] and Damage-specific DNA Binding protein I [56] are involved in the repair of DNA lesions, and were clustered by DAVID into the nucleotide repair pathways that had no fingerprints of positive selection. Seldomly occurring, some amino acid replacements can lead to changes in proteins that are advantageous in new environments and selected for and kept in the species lineage.

Our branch-sites model selection test identified 12 positively selected genes in the *Holbrookia* lineage that have roles in immunity, development, and metabolism. From the twelve positively selected genes, three genes, *BNIP3L*, *LBHD2*, and *PHGDH*, are of particular interest for their potential role in shaping morphology, resistance against viruses, and amino acid production. The *LBH* (Limb-bud-and-heart) Domain-2 is part of a protein family that are key transcription regulators in embryonic development. The *LBH* gene is expressed early in embryogenesis with proteins playing a role in the development of limb buds and heart formation in a mice-model system [57]. A previous study has identified a single nonsynonymous mutation in an *LBH* homolog [58] associated with an adaptive variation, and here we have found multiple amino acid replacements selected for in the *Holbrookia* lineage. While we do not know the functional consequences of these amino acid changes, they may play an important role in local adaptation to desert and grassland environments that *Holbrookia* inhabit.

Immune genes are known hotspots for selection to act on, having persistent selective pressures by pathogens that elicit immune responses. *BNIP3L* promotes apoptotic activity, targeting dysfunctional mitochondria organelles. Specific viruses produced anti-apoptotic proteins that suppress the apoptosis of virally infected cells, allowing viruses to replicate and multiply. *BNIP3L* proteins interact with viral and cellular anti-apoptosis proteins and overcome the suppression to initiate apoptosis [59,60].

Amino acids are the essential building blocks to protein, and here we found the *PHGDH* (D-3-phosphoglycerate dehydrogenase) gene that has a role in amino synthesis has experienced positive selection with multiple amino acid replacements. The protein encoded by this gene is responsible for producing the non-essential amino acid L-serine. While described as non-essential, L-serine is a critical precursor required for the synthesis of D-serine, amino acids, and other metabolites in animal cells [61]. Deficiencies in the D-3-phosphoglycerate dehydrogenase protein results in metabolic defects affecting the nervous systems [62,63]. The kidneys produce the majority of L-serine under normal conditions [61]. When dietary protein is limited, the liver becomes the primary production of the metabolite. While this has been shown only in mammals, it is of interest to see if a similar pattern will be observed in *Holbrookia* species as the alteration of grassland for agriculture purposes and the use of pesticides, can impact the invertebrate populations and reduce their food availability.

## Conclusions

While there are still problems in conservation genetics that can still be answered successfully using conventional conservation genetics techniques, the low cost of HTS technologies allows us to address questions of genetic diversity, adaptation, and taxonomy. High-throughput sequencing technologies are improving on traditional approaches generating extensive molecular resources for organisms of conservation interest. Scaling up from just a few loci to genomics and transcriptomics allows for better inferences and conservation practices. Here we have generated the first transcriptome for three lizards in the genus *Holbrookia* and identified multiple genes under positive selection. These transcriptomes have already provided insight into potentially adaptive loci in *Holbrookia* and will continue to contribute to future population-based and systematics studies of iguanian lizards.

## Supporting information

S1 Table (XLSX)

S1 Data (TXT)

S1 File (PHY)

S2 File (PHY)

S3 File (PHY)

S4 File (PHY)

S5 File (PHY)

S6 File (PHY)

S7 File (PHY)

S8 File (PHY)

S9 File (PHY)

S10 File (PHY)

S11 File (PHY)

S12 File (PHY)
